# A synthetic biology standard for Chinese Hamster Ovary cell genome monitoring and contaminant detection by polymerase chain reaction

**DOI:** 10.1186/s40064-016-3074-8

**Published:** 2016-09-08

**Authors:** Alexander Templar, Douglas Marsh, Darren N. Nesbeth

**Affiliations:** Department of Biochemical Engineering, University College London, Bernard Katz Building, London, WC1E 6BT UK

**Keywords:** PCR, Synthetic biology, Standardisation, Mammalian cells

## Abstract

**Background:**

Chinese Hamster Ovary (CHO) cells are the current industry standard for production of therapeutic monoclonal antibodies at commercial scales. Production optimisation in CHO cells hinges on analytical technologies such as the use of the polymerase chain reaction (PCR) to quantify genetic factors within the CHO genome and to detect the presence of contaminant organisms. PCR-based assays, whilst sensitive and accurate, are limited by (i) requiring lengthy sample preparation and (ii) a lack of standardisation.

**Results:**

In this study we directly assess for the first time the effect of CHO cellular material on quantitative PCR (qPCR) and end-point PCR (e-pPCR) when used to measure and detect copies of a CHO genomic locus and a mycoplasma sequence. We also perform the first head-to-head comparison of the performance of a conventional qPCR method to that of the novel linear regression of efficiency (LRE) method when used to perform absolute qPCR on CHO-derived material. LRE qPCR features the putatively universal ‘CAL1’ standard.

**Conclusions:**

We find that sample preparation is required for accurate quantitation of a genomic target locus, but mycoplasma DNA sequences can be detected in the presence of high concentrations of CHO cellular material. The LRE qPCR method matches performance of a conventional qPCR approach and as such we invite the synthetic biology community to adopt CAL1 as a synthetic biology calibration standard for qPCR.

## Background

Design and construction of synthetic eukaryotic genomes (Dymond et al. 2011) has made rapid progress in recent years, alongside conventional recombinant DNA approaches to construction of human artificial chromosomes (Kononenko et al. 2015). It is logical to expect these two research trajectories will converge with the design, construction and implementation of synthetic genomes to control mammalian cells. This holds the promise of powerful control of those mammalian cell characteristics that currently limit their performance in industrial settings. One challenge raised by this approach is the need for standardised assays to quantify the presence or loss of operationally critical genetic elements within a synthetic genome. Standardisation of data capture metrics is a defining feature of both synthetic biology and industrial bioprocessing and is critical to reproducible manipulation of host chassis (Kitney and Freemont 2012). Data captured during industrial scale cell cultivation is also essential to achieve process understanding, which in turn is necessary for optimisation of product yield and quality (Clementschitsch and Bayer 2006).

Mammalian cells have the capacity to produce recombinant proteins with human or near-human protein glycosylation patterns (Walsh and Jefferis 2006), a feature essential for effective production of monoclonal antibodies (mAbs), which represent more than half the products in the rapidly growing biopharmaceutical industry (Ecker et al. 2015). The Chinese Hamster Ovary (CHO) cell production platform is one of the most widely researched and exploited chassis for the production of biologics such as mAbs (Kim et al. 2011). Mycoplasmal infections of CHO and other mammalian cells types can distort cell phenotype, compromise host genome integrity (Lincoln and Gabridge 1998) and confound efficacy of cell-based therapies (US Food and Drug Administration and Center for Biologics Evaluation and Research 2010). As such mycoplasmal infection is a major risk factor that potentially jeopardises clinical translation of many of the exciting advances made by synthetic biologists in areas such as T cell therapy.

Due to its high sensitivity and exquisite accuracy (Bartlett and Stirling 2003), the polymerase chain reaction (PCR) has proven to be a powerful platform for bioprocess analysis. A number of applications exist for PCR-based assays including measurement of mycoplasma infection (Falagan-Lotsch et al. 2015), genetic drift (Voronin et al. 2009), community modelling (Tolvanen et al. 2008), cell barcoding and identification (Parodi et al. 2002) and quantitation of process stream contamination (Barker et al. 2010; Uphoff and Drexler 2011). Future avenues for the production of biologics, such as those that will employ chassis with fully or largely synthetic genomes (Dymond et al. 2011), suggests the applicability and relevance of PCR-based sequence specific nucleic acid analysis is set to increase.

The most commonly reported use of real-time PCR is relative quantitative PCR (qPCR) that provides the ratio of two mRNA transcripts within a given sample, typically a reference gene and a gene of unknown expression level (Nolan et al. 2006). Reverse transcriptase (RT) is used to convert the mRNA transcripts to single stranded complementary DNA (cDNA) molecules. Subsequent PCR amplification from cDNA template can be recorded in real time by the appearance of fluorescence as a result of a fluorescent dye binding to the double stranded DNA (dsDNA) PCR product (amplicon). The number of PCR cycles required for the fluorescence level to pass a set threshold is known as the quantification cycle (Cq) number and can be related back to the amount of template present in the sample. Although relative RT-qPCR is a powerful tool for basic research, absolute quantification using measures that can ideally be converted to internationally recognised units is always the preferred option for engineering and, by extension, synthetic biology.

The absolute measurement preferred by engineers can be achieved using qPCR when a standard curve (SC) is used for calibration (Pfaffl et al. 2002). Such standard curves are made using a purified stock, of known concentration, of the template DNA for which the concentration in the experimental samples is unknown. This purified stock is then serially diluted and each dilution of template used for an individual PCR. The resultant Cq values for each PCR can be related to the known mass of starting template DNA per sample using a statistical treatment that assumes the reaction efficiency for each dilution has proceeded with equal efficiency. Subsequent experiments are then calibrated against this standard curve.

Rutledge and Côté (2003) observed that in fact PCR efficiency is rarely equal across the samples of a dilution series and in response developed the Linear Regression of Efficiency method of absolute qPCR (LRE qPCR). LRE qPCR exploits the fact that the signal from the fluorescent, dsDNA-binding dye, SYBR Green I, is not influenced by amplicon size or sequence (Spandidos et al. 2008) and that a Boltzmann sigmoidal statistical framework can be applied directly to PCR amplification profiles such that Cq determination is not needed (Rutledge and Stewart 2008). The direct linkage of base pair formation to absolute fluorescence units (FU) makes LRE qPCR a strong candidate as a method synthetic biologists could use to obtain absolute, rather than relative, measurements of specific DNA sequences. Using absolute FU also enables the selection of a universal calibration standard. Rutledge and Stewart (2010) investigated a selection of primer and template combinations, from which the CAL1 reaction proved optimal in terms of performance and reliability as a universal standard.

Currently, PCR-based assays have few widely adopted standardised elements, with individual laboratories and facilities each using their own assay methodologies and oligonucleotide sequences for assay calibration. Furthermore, sample preparation procedures that precede PCR assays typically extend assay throughput time, increase labour and can introduce error (Skulj et al. 2008). This presents a particular limitation for industrialists seeking to gain insight from approaches such as process analytical technology (PAT) which ideally require real-time or at-line analysis (Kaiser et al. 2008).

With these challenges in mind we suggest it is prudent to define some of the factors involved in developing rapid, industrially robust and standardised PCR-based assays for monitoring of both CHO cell genomic loci and contaminant organisms known to be a risk factor in CHO cultivation. To do this we quantitate the impact of CHO cells, from shake flask and bioreactor cultivation, on the performance of PCR. We test end point PCR (e-pPCR), a conventional method of standard curve-based (Pfaffl et al. 2002) quantitative PCR (SC qPCR) and the recently-developed linear regression of efficiency qPCR method (LRE qPCR), which features the CAL1 calibration reaction (Rutledge and Stewart 2010) reported to have ideal amplification properties that enable its use as a universal standard. We believe the resulting data will reveal the extent to which sample preparation is in fact required for PCR, if at all, enabling future efforts to develop a rapid, robust and standardised PCR assay. We also examine our findings in light of possible application of the LRE qPCR calibration reaction as a synthetic biology standard.

## Methods

### Materials

All reagents were of molecular biology grade unless otherwise stated. All stocks, solutions and reagents were prepared or brought to volume with the Millipore (Billerica, USA) ‘Water for Molecular Biology’ product which is confirmed as DNA and RNAse free by the supplier. All oligonucleotides were synthesised by Eurofins MWG Operon (Acton, UK, www.eurofinsdna.com).

### Cultivation of CHO cells

A clonally derived glutamine synthase (GS) CHO cell line stably expressing an antibody-based therapeutic was cultivated in 1L Erlenmeyer shake flasks (SF) according to the protocol described by Velez-Suberbie et al. (2013) until a viable cell count of 2.5 × 10^6^ cell/mL was reached, as determined by ViCell-XR cell viability analyser (Beckman Coulter, USA). At this point a sample was taken for further PCR experiments (Fig. 2a). This stage represents a critical point of industrial scale cultivation where the seed train is used to inoculate the larger scale growth vessel. The shake flask culture was added to CD-CHO media in a rocked bag bioreactor to an initial concentration of 2.5 × 10^5^ cells/mL. Bioreactor cultivation was performed in a 3L Applikon Appliflex (Applikon, Holland) flexible rocked bag bioreactor, controlled by an Applikon EZ controller system. Temperature was kept at 37 °C with the dissolved oxygen (DO) set-point at 30 % and the pH set-point at 7.1 ± 0.05. Glucose concentration was maintained at 150 g/L, as determined by NOVA Bioanalyser 400 (NOVA Biomedical, Waltham, USA), by supplementing with 10× concentrated CD-CHO media (Life Technologies, Paisley, UK). Cells grew to achieve 1 × 10^7^ cells/mL with ~99 % viability (Fig. 2b) in the bioreactor and a sample was removed for PCR.

### Nucleic acid purification

DNA was purified as detailed below from the shake flask (Fig. 2a) and wave-bag bioreactor (Fig. 2b) samples to determine typical DNA measurements by spectrophotometry. After this scoping study, the volume of sample, ranging from 1.6 to 6.5 mL, required to provide the DNA concentration in the undiluted template reactions indicated in Figs. 3 and 4, was centrifuged at 10,000 RPM for 3 min, re-suspended in 400 µL of lysis buffer (2 % Triton X100, 1 % SDS, 100 mM NaCl, 10 mM Tris–HCl, 1 mM EDTA) and freeze-thawed twice by incubating at −80 °C for 3 min and 95 °C for 1 min. Total nucleic acid was purified using standard phenol/ethanol extraction procedure and resuspended in 400 µL 10 mM Tris buffer (pH 7.5). Six aliquots of purified DNA were made and stored at −20 °C. A given aliquot was thawed once for experimentation and any unused portion of the aliquot discarded. The proxy plasmid pPROX2 was purified with a Key Prep ‘mini prep’ kit (Anachem, Luton, UK).

### Cell disruption

**Fig. 1:**
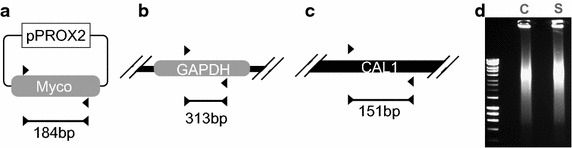
Gel analysis of gDNA and overview of polymerase chain reactions in this study. Primers (*black triangles*) detailed in Table 2 were used to amplify a mycoplasma sequence present in the plasmid pPROX2 (3010 bp) as a proxy for pathogen detection (**a**), target DNA within the mammalian GAPDH gene in the CHO genome (**b**) and the designated CAL1 locus with the lambda phage genome (**c**). Expected amplicon size (bp) is indicated under the bar at the bottom of each panel. (**d**) 60 µL of a 2.5 × 10^6^ cell/mL cell suspension was ran on a gel before sonication in the *lane* marked ‘*C*’. 60 µL of a 2.5 × 10^6^ cell/mL cell suspension was ran on a gel after sonication in the *lane* marked ‘*S*’. Molecular weight ladder was run in *leftmost lane*, uppermost band is 10 kilo-base pairs

**Fig. 2:**
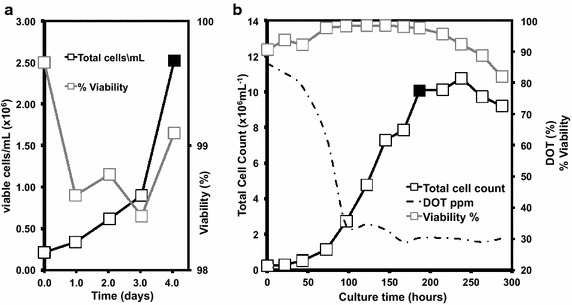
Cultivation of CHO cells. Growth profile of CHO cells in 1L Erlenmeyer flasks (**a**) and in an Applikon 3L rocked bag bioreactor fermentation (**b**). Samples for PCR experiments were taken at the time points indicated (*closed symbols*)

**Fig. 3:**
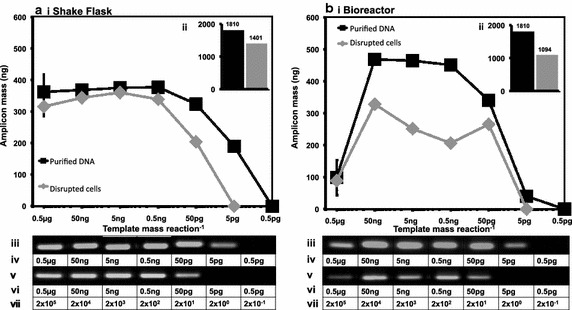
Influence of disrupted CHO cells on e-pPCR detection of a genomic target sequence. Disrupted cells and purified DNA from samples taken from shake flask (**a**) and bioreactor (**b**) cultivation were used as template material for e-pPCR. For both cultivation methods the following data are depicted. The mass of amplicon produced in a reaction is plotted as a function of sample dilution (*i*). Inlaid graphs (*ii*) plot the area (arbitrary units) under each curve as a bar chart. For both graphs, agarose gel images show the amplicon band generated from the purified DNA (*iii*) and disrupted cell samples (*v*). Template DNA mass in disrupted cell samples (*vi*) was estimated by spectrophotometry and densitometry. Template DNA mass in purified DNA samples was also estimated in this way (*iv*). From this mass the predicted copy number (*vii*) of genomes ranges from 1.89 × 10^5^ (rounded to 2 × 10^5^ in the graphic), in the undiluted 0.5 µg samples, to 0.189 (rounded to 2 × 10^−1^ in the graphic), in the 0.5 pg tenfold diluted samples

**Fig. 4:**
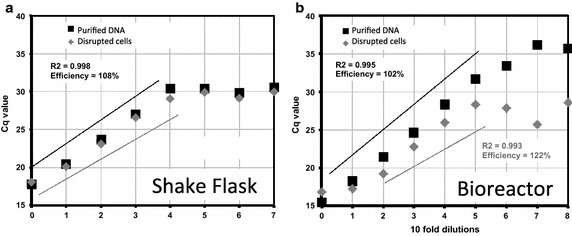
Influence of disrupted CHO cells on amplification efficiency for a genomic target. Real time PCR was performed using disrupted cells (*grey symbols* and *lines*) or purified DNA (*black symbols* and *lines*) from shake flask (**a**) and bioreactor (**b**) cultivation as template. Undiluted shake flask cell-sonicate and purified DNA samples were estimated to contain 115 gDNA (5.1 × 10^4^ genome copies) by spectrophotometry and densitomery. Undiluted bioreactor cell-sonicate and purified DNA samples were estimated to contain 1 µg gDNA (3.78 × 10^5^ genome copies) by spectrophotometry and densitomery. C_q_ values were plotted against tenfold dilutions of template source. *Lines* indicate data points for which amplification efficiency is 100 ± 10 % efficiency, at a confidence level of R^2^ > 0.99. Data featured is typical of n = 3 analytical repeats

Cell suspensions from the shake flask (Fig. 2a) and wave-bag bioreactor (Fig. 2b) samples were sonicated as detailed below to determine typical DNA estimations by spectrophotometry and densitometry. After this the volume of sample, ranging from 1.6 to 6.5 mL, required to provide the DNA concentration in the undiluted cell sonicate template reactions indicated in Figs. 3 and 4, was centrifuged at 10,000 RPM for 3 min and re-suspended in 400 µL dH_2_O. A Soniprep 150 sonicator (MSE, London, UK) was used to subject samples to three repeats of the following procedure: 10 s cycles of 100 % amplitude sonication followed by 10 s rest, for a total duration of 60 s. 60 µL of a 2.5 × 10^6^ cell/mL cell suspension from shake flask cultivation was ran on a standard 1 % w/v agarose gel before sonication and 60 µL ran after sonication. Figure 1 shows the gel and the same pattern was observed for cells cultivated in bioreactors.

### Mycoplasmal DNA sequence

A 3010 bp plasmid, pPROX2, was designed to encode 300 bp of a 16 s RNA signature gene (Table 1) present in five species of *Mycoplasma* that are commonly found in infected mammalian cell culture (Kong et al. 2001). The gene segment was inserted into a pUC57 plasmid by Eurogentec (Liege, Belgium) and the plasmid propagated using standard molecular biology techniques.

**Table 1:** 300 bp sequence inserted into plasmid

*Mycoplasma* spp. 16s RNA sequence	
TTGTACTCCGTAGAAAGGAGGTGATCCATCCCCACGTTCTCGTAGGGATACCTTGTTCGACTTAACCCCAGTCACCAGTCCTGCCTTAGGCAGTTTGTTTATAAACCGACTTCGGGCATTACCAGCTCCCATGGTTTGACGGGCGGTGTGTACAAGACCCGAGAACGTATTCACCGTAGCGTAGCTGATCTACGATTACTAGCGATTCCGACTTCATGTAGTCGAGTTGCAGACTACAATCCGAACTGAGACCGGTTTTTTGAGGTTTGCTCCATGTCACCACTTCGCTTCTCTTTGTA	

### PCR primer design

Primer sequences (Table 2) were designed in accordance with ‘minimum information for publication of quantitative real-time PCR experiments’ (MIQE) guidelines (Bustin et al. 2009) and screened in silico for specificity and potential for self-annealing using the NCBI primer blast tool (http://www.ncbi.nlm.nih.gov/tools/primer-blast/, accessed 22.05.15) and the PCR primer stats tool (http://www.bioinformatics.org/sms2/pcr_primer_stats.html, accessed 22.05.15) respectively. We designed a plasmid, pPROX2, encoding 300 bp of mycoplasma DNA (Table 1) as a safe alternative to using live or attenuated mycoplasma in a research facility also used for large scale mammalian cell cultivation. Whilst there are 20 species of mycoplasma known to infect mammalian cell culture, six species are identified in most infections. The 300 bp sequence is conserved across five of the six mycoplasmas common to 90–95 % of mammalian cell culture infections (Kong et al. 2001). The glyceraldehyde 3-phosphate dehydrogenase (GAPDH) locus (Gene ID: 100736557) of the CHO cell genome was chosen as single copy genomic target (Table 1; Fig. 1). The CAL1 primers (Table 2; Fig. 1) defined by Rutledge and Stewart (2010) were used for LRE qPCR. Agarose gel electrophoresis showed all three reactions produced only amplicon of expected size.

**Table 2:** Oligonucleotides used in PCR

CHO GapDH gene (GeneID: 100736557)	GapDH Fwd	CATCACCATCTTCCAGGAGC
	Rev GapDH	CTTGGTTCACACCCATCACA
Conserved mycoplasma gene	Myco Fwd	AAACCGACTTCGGGCATTAC
	Rev Myco	GAAGTGGTGACATGGAGCAA
CAL1 primers	Cal 1 Fwd	AGACGAATGCCAGGTCATCTGAAACAG
	Rev CAL1	CTTTTGCTCTGCGATGCTGATACCG

### Preparation and analysis of material containing template DNA for PCR

To evaluate the effect of cellular material on PCR assay performance we disrupted cells using the gentle sonication procedure detailed above which ensured gDNA remained largely intact and was not denatured to any significant degree. This was confirmed by agarose gel electrophoresis of cell suspensions before and after sonication (Fig. 1). Disrupted cell samples were compared to samples in which total nucleic acids had been isolated using standard phenol–chloroform extraction. Spectrophotometry was used to determine DNA mass to enable genome copy number estimation by a method that is mechanistically unrelated to PCR. Three spectrophotometric measurements were taken over three tenfold serial dilution, and this was used to predict DNA mass over further dilution. Densitometric analysis of gel images was also used to estimate total DNA concentration present in a given sample of disrupted cells.

### End-point PCR

Reactions were carried out in a total volume of 50 µL, with 5 µL of 10× MgCl_2_ polymerase buffer (100 mM Tris/HCl, 15 mM MgCl_2_, 500 mM KCl), 0.5 µL Taq polymerase, 1 µL 10 mM dNTP (Sigma Aldrich, St. Louis, MO, USA), 5 µL of material containing template DNA and 2.5 µL of primer at a concentration of 1 µM (to give a final concentration of 500 nM of each primer per reaction). A Veriti 96 well thermocycler (Applied Biosystems Grand Island, NY, USA) was used with a cover heated to 105 °C. Each PCR was run for 40 cycles of: 95 °C for 5 s, 57 °C for 5 s, 72 °C for 30 s.

### Quantitative PCR

**Fig. 5:**
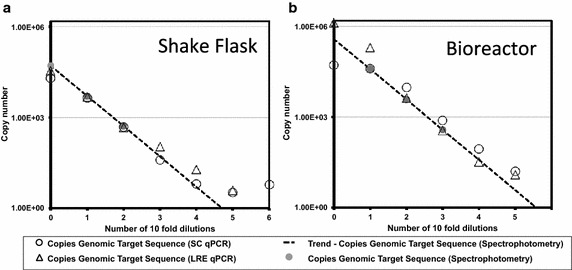
Qualitative comparison of SC qPCR and LRE qPCR for quantitation of a genomic sequence. The same samples as used in Fig. 4 were used to assess LRE qPCR (*open triangles*) and SC qPCR (*open circles*) performance. Copies of the GAPDH target sequence present in a sample were measured by each method and plotted as a function of sample dilution for samples derived from shake flask (**a**) and bioreactor (**b**) cultivation. *Grey circles* indicate genome copy number inferred from a spectrophotometric measurement of total DNA concentration present in sample. The *dashed lines* indicate linear extrapolation of the spectrophotometric data points

Reactions were carried out in a total volume of 20 µL, with each reaction containing 10 µL of 2× SsoAdvanced SYBR Green Supermix (BioRad, Hercules, CA, USA), 5 µL of material containing template DNA and 1 µL of primer at a concentration of 1 µM (to give a final concentration of 500 nM of each primer per reaction). Reactions were performed in a CFX Connect Real-time PCR Detection System (Bio-Rad, Hercules, CA, USA) with a cover heated to 105 °C. Each reaction was run at a total of 40 cycles, with the same cycling conditions as above. For qPCR experiments plotted in Figs. 4, 5, 7, 9 and 10, each sample was split into three and amplified in separate wells of a 96 well plate. The average of the resultant Cq values formed the Cq value or copy number value and the standard deviation of all three was used to plot error bars. These error balls always fell within the areas of the symbols or lines used to indicate data points.

**Fig. 6:**
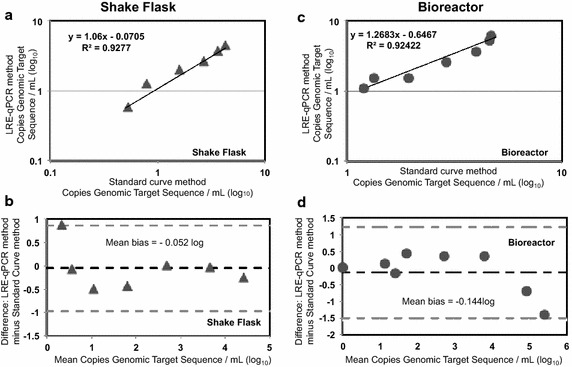
Statistical comparison of SC qPCR and LRE qPCR for quantitation of a CHO genomic sequence. Figure 5 data from SC qPCR and LRE qPCR methods to measure GAPDH copies in shake flask and bioreactor samples were compared using XY plot, graphs **a**, **c** respectively, and Bland–Altman plot, graphs **b** and **d** respectively. Statistical procedures were performed as described by Burd (2010). The mean bias (overall average difference) is indicated by the *dark dashed line* and 1.96× the standard deviation (±) of this bias is indicated by the *grey dashed lines*, to show the limits within which bias levels have a 95 % confidence interval

**Fig. 7:**
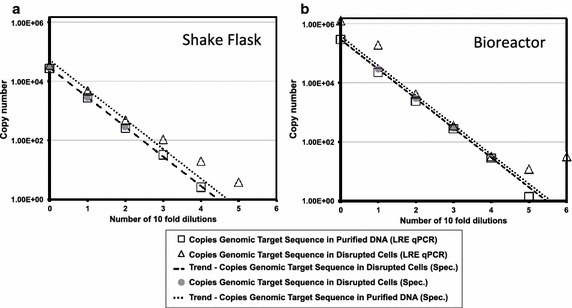
Influence of disrupted CHO cells on LRE qPCR for quantification of a CHO genomic target. The same samples as used in Fig. 4 were used to assess the effect of cellular material on LRE qPCR performance. The predicted number of copies of the GAPDH target sequence in a sample, as calculated using the LRE qPCR method, were plotted as a function of sample dilution for samples derived from shake flask (**a**) and bioreactor (**b**) cultivation. Samples either underwent total DNA purification (*open squares*) or only mild cell disruption (*open triangles*) prior to LRE qPCR procedure. Genome copy number was also inferred from spectrophotometric measurement of total DNA concentration. These spectrophotometric measurements are indicated by *large grey circles* and are linearly extrapolated for both purified DNA (*thick dashed line*) or disrupted cell (*fine dashed line*) samples

**Fig. 8:**
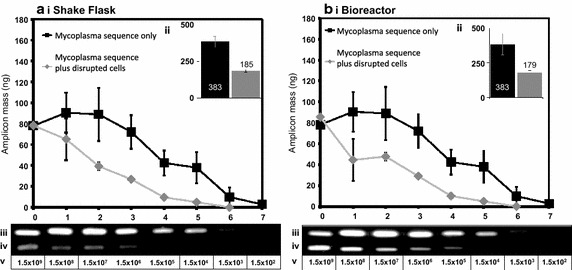
Influence of disrupted CHO cells on e-pPCR detection of a mycoplasmal target sequence. 5 ng of plasmid encoding a mycoplasmal DNA sequence (1.54 × 10^9^ copies) was used as e-pPCR template either as purified DNA or purified DNA plus disrupted cells derived from a sample containing a total of 2 × 10^6^ cells from shake flask (**a**) and 2.5 × 10^5^ cells from bioreactor (**b**) cultivation. For both cultivation methods the following data are depicted. The mass of amplicon produced in a reaction is plotted as a function of sample dilution (*i*). Inlaid graphs (*ii*) plot the area (arbitrary units) under each *curve* as a *bar chart*. Agarose gel images show the 184 bp amplicons generated from the purified plasmid DNA (*iii*) and plasmid DNA plus disrupted CHO cells (*iv*). The number of copies of the plasmid molecule in a given sample is indicated in the row labelled (*v*)

**Fig. 9:**
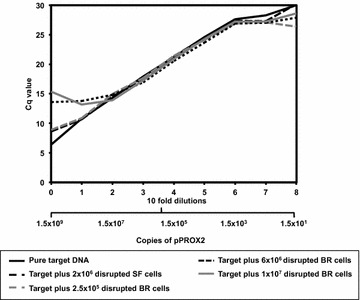
Influence of disrupted CHO cells on amplification efficiency for a mycoplasmal target sequence. Real time PCR was performed using 5 ng (1.54 × 10^9^ copies) of plasmid encoding a mycoplasmal DNA sequence and a further 8 tenfold dilutions of the plasmid template. For each starting solution either zero cells were present or disrupted cells derived from samples of 2 × 10^6^ cells/mL, from shake flask cultivation, or 2.5 × 10^5^, 6 × 10^6^ and 1 × 10^7^ cells/mL, from bioreactor cultivation, were added to plasmid DNA (see *legend*). The resultant Cq values for each amplification reaction were plotted as a function of sample dilution. Data featured is typical of N = 3 analytical repeats

**Fig. 10:**
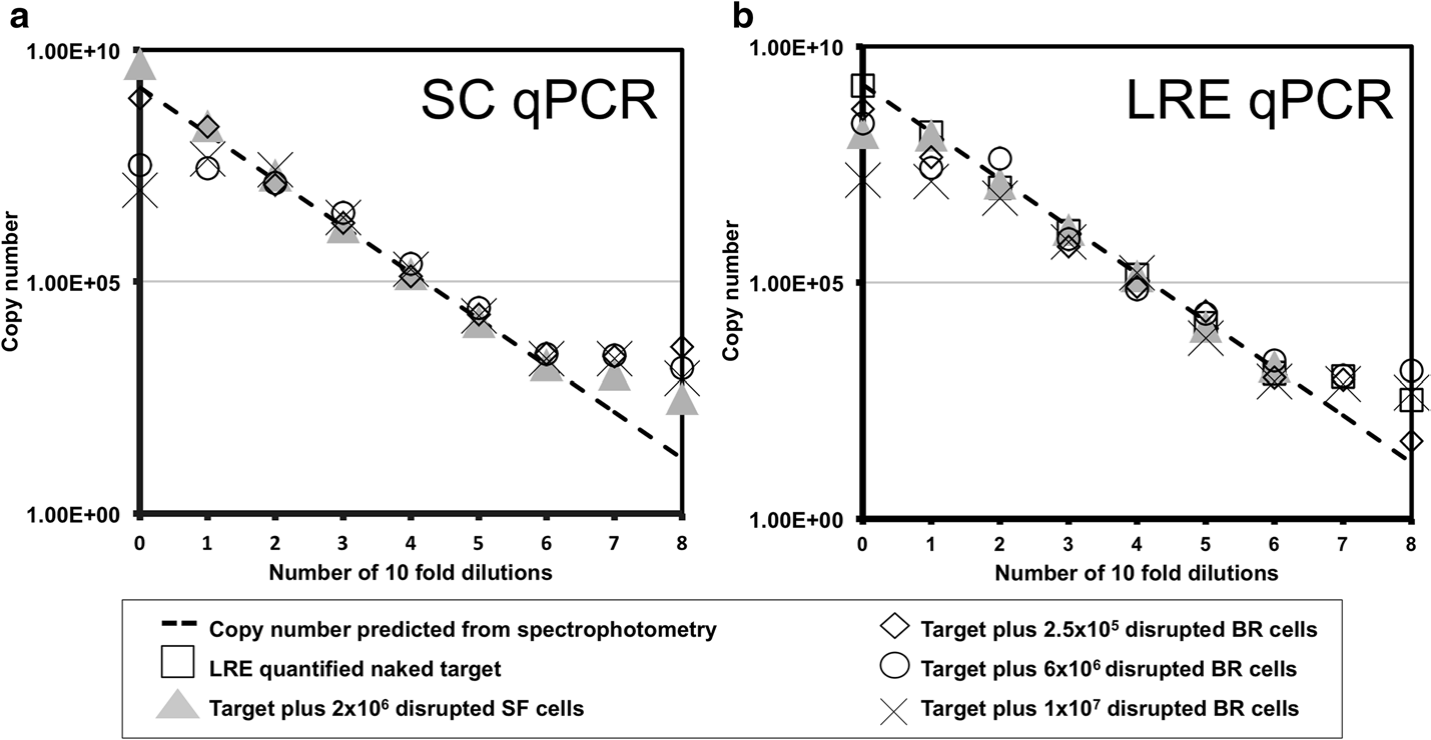
Comparison of SC qPCR and LRE qPCR methods for absolute quantitation of a mycoplasmal DNA sequence. SC qPCR (**a**) and LRE qPCR (**b**) methods were used to quantify the number of copies of a mycoplasmal DNA sequence present in reactions containing 5 ng plasmid DNA (1.54 × 10^9^ copies) plus disrupted CHO cells derived from a 2 × 10^6^ cells/mL shake flask sample or 2.5 × 10^5^, 6 × 10^6^ and 1 × 10^7^ cells/mL bioreactor cultivation samples (see *legend*). The number of copies of the plasmid in a given sample was also inferred from spectrophotometric measurement of total DNA concentration before addition of disrupted cells. These spectrophotometric measurements (not indicated for clarity) are linearly regressed (*thick dashed line*) for qualitative comparison. For LRE qPCR (**b**) it is possible to assess quantification of the pure mycoplasmal sequence (*open squares*) because the unrelated CAL1 reaction is used for calibration. For SC qPCR (**a**), quantification of the pure mycoplasmal sequence is not informative as this reaction represents the standard curve used for calibration

### Reaction efficiency analysis

For end-point PCR, the area under the curve was calculated using the trapezoidal method (Atkinson 1978). For SYBR-green labelled PCR, Cq values were generated using Bio-Rad CFX manager 3.0 (BioRad, Hercules, CA, USA), then exported into Microsoft Excel 2010 for analysis. Efficiency was calculated with the standard curve method (Rutledge and Stewart 2008) from a trend-line, drawn between points above a minimum R^2^ threshold of 0.99. Linear regression was then applied to calculate efficiency (E), with the equation:

$$ E = 10^{{\left( {\frac{ - 1}{slope}} \right)}} $$

### Copies of target DNA determined by Standard Curve and Cq values

The standard curve generated as described above was used to estimate copies of target in samples containing cell debris. Cq values of cell debris samples were plotted along the standard curve and converted into copy number using the equation:

$$ target\; copy\; number = 10^{{\left( {\frac{Cq - b}{m}} \right)}} $$

where b is the y-intercept and m is the slope of the standard curve.

### Copies of target DNA determined by LRE-qPCR

LRE-qPCR, as described by Rutledge and Stewart (2008), was also applied to estimate copy numbers. LRE analyser v. 0.97 (Rutledge 2011) was used according to developers instructions. A dilution set of pure lambda DNA (product code N3011S from New England BioLabs, Ipswich, MA, USA) was used with the CAL1 primer pair to calibrate the data.

## Results

### CHO cellular material reduces e-pPCR sensitivity tenfold for detection of a genomic locus

As well as contaminant detection, end-point PCR (e-pPCR) is widely used to confirm the identity of a host cell by confirming the presence of a single genomic locus (Parodi et al. 2002). We quantified the impact of disrupted cells, from shake flask and 3 L scale bioreactor cultivation, on the limit of detection (LOD) for e-pPCR used to confirm the presence of a sequence within the single genomic copy GAPDH gene. Template material consisted of either 500 ng (1.89 × 10^5^ genome copies) genomic DNA purified from a cell suspension sample, or 2 × 10^5^ disrupted cells containing the same mass of genomic DNA. LOD was taken to be the first tenfold dilution of template material for which no amplicon band could be detected after n = 3 experimental repeats. The presence of disrupted cells raised e-pPCR LOD tenfold, from 0.2 genome copies to 2 genome copies, for both shake flask (Fig. 3a) and bioreactor (Fig. 3b) derived material. Marginally lower overall amplicon production was observed in samples taken from bioreactor cultivation (Fig. 3b-ii) compared to shake flask (Fig. 3aii).

### Efficiency of genomic target amplification is reduced by cellular material from bioreactor cultivation

Numerous methods have been defined to assess the accuracy of qPCR. A common approach is to consider the efficiency of amplicon production as an indicator of fidelity. Efficiency is calculated by plotting Cq value as a function of decreasing template mass (Rutledge and Côté 2003). 100 ± 10 % efficiency, at a confidence level of R^2^ > 0.99 is typically set as the threshold for amplification to be considered accurate. We determined the impact of disrupted cells from shake flask cultivation by measuring the amplification efficiency for the GAPDH target using either 115 ng gDNA (5.1 × 10^4^ genome copies) purified gDNA, or suspensions of 5.5 × 10^4^ disrupted cells, determined by spectrophotometry to contain the same DNA mass (Fig. 4a), as template material. For bioreactor cultivation 1 µg purified gDNA (3.78 × 10^5^ genome copies) and 4 × 10^5^ disrupted cells, determined by spectrophotometry to contain the same DNA mass, was used as template (Fig. 4b). Disrupted cells from shake flask cultivation had no marked impact on the efficiency profile for the reaction (Fig. 4a). By contrast cellular material from bioreactor cultivation constricted the window of efficient amplification from six tenfold dilutions for pure DNA template down to four when disrupted cells are present (Fig. 4b).

### LRE qPCR is equivalent to SC qPCR with respect to quantification performance for a CHO genomic target

Two methods of quantitation, the traditional standard curve (SC) qPCR approach and the recently developed method of LRE qPCR (Rutledge and Stewart 2008), were used for absolute qPCR analysis of the same disrupted cell samples used in Fig. 4. LRE qPCR was calibrated using the CAL1 primers and methods detailed by Rutledge and Stewart (2010). Both methods were compared to copy numbers derived from spectrophotometric measurements (dotted lines in Fig. 5). Assuming a genome size of 2.45 Gb, four or more tenfold dilutions of the starting material from shake flask cultivation (Fig. 5a) should result in samples containing less than 5 copies of the CHO genome. As such it is not surprising that after four tenfold dilutions of the initial sample both SC and LRE qPCR data diverge from projections based on spectrophotometry (Fig. 5a).

For bioreactor-derived material (Fig. 5b), LRE qPCR data largely agreed with spectrophotometry data over dilutions 2–4, after which LRE qPCR data flattened for samples predicted to contain five copies or less of the CHO genome. Overall, for bioreactor-derived cellular material the LRE qPCR data matches spectrophotometric projections more closely than SC qPCR over almost every sample dilution (Fig. 5b).

Method comparison by XY plot (Burd 2010) gives a slope of 1.00 and an intercept of zero in the case of zero proportional bias between methods. For shake flask-derived material an XY plot (Fig. 6a) showed negligible proportional bias of SC qPCR data (slope of 1.06) when using the LRE qPCR method. The Y intercept of the XY plot was close to zero (0.0705) indicating little systematic bias. A Bland–Altman (Bland and Altman 1986) plot (Fig. 6b) indicates LRE qPCR exhibited a positive bias of SC qPCR at higher copy numbers of target DNA but that LRE qPCR and SC qPCR are broadly equivalent due to the fact that the mean bias range for both methods includes zero difference (Burd 2010).

For qPCR of bioreactor-derived material an XY plot (Fig. 6c) showed that LRE qPCR exhibited significant proportional (slope of 1.26831) and systemic (intercept of 0.6467) bias of SC qPCR data. Bland–Altman plot (Fig. 6d) indicated LRE qPCR had negative bias of SC qPCR data at high target DNA copy number but that the methods are statistically equivalent as the bias range spans zero difference.

### LRE qPCR quantitation of genomic target is largely unaffected by CHO cellular material

LRE qPCR is calibrated from an external lambda DNA sample that involves direct analysis of fluorescence data so we were able to use this method to quantify target in purified DNA samples as well as in disrupted cell solutions. In this way we could use the same samples used in Fig. 4 to evaluate the effect of cellular material on LRE qPCR performance. For purified DNA samples derived from shake flask cultivation, Fig. 7a shows that LRE qPCR (open squares) agrees well with spectrophotometric data (thick dashed line). The presence of disrupted cells from shake flask cultivation caused divergence between LRE qPCR data (open triangles) and spectrophotometric data (fine dashed line) when 5 or less genome copies are predicted to be present in the sample. The equivalent profile for bioreactor-derived samples (Fig. 7b) was broadly the same except the presence of disrupted cells caused LRE qPCR data to diverge from spectrophotometric data when the number of genome copies predicted to be present was 4 or less or 40,000 and above.

### Sensitivity of e-pPCR for mycoplasma DNA sequence detection is depressed by CHO cellular material

A common application of ePCR is the binary detection of organisms known to contaminate cultures of mammalian cells at industrial scale. As such we designed the 3010 bp pPROX2 plasmid containing a 300 bp sequence conserved across five species of mycoplasma (Kong et al. 2001) and used this as a safe proxy test of the sensitivity of e-pPCR for mycoplasma detection. We used a mycoplasma genomic locus sequence present in pPROX2 as this afforded us (i) exquisite control over the copy number of the gene achievable by serial dilution into our samples and (ii) a safer option than using material directly derived from mycoplasma which could possibly be contaminated with live mycoplasma cells. Serial dilutions of a solution containing 5 ng (1.54 × 10^9^ copies) of the pPROX2 plasmid were made and to each dilution a constant volume of either water or disrupted cells was added (Fig. 8). Disrupted cells were generated from a sample containing 2 × 10^6^ cells/mL from shake flask cultivation (Fig. 8a) or from a sample of 2.5 × 10^5^ cells/mL from bioreactor cultivation (Fig. 8b). The LOD for naked DNA template was 154 copies (0.5 fg pDNA). This was increased tenfold to 1540 copies (5 fg pDNA) by the presence of disrupted cells, from either shake flask (Fig. 8a) or bioreactor (Fig. 8b) cultivation. Total amplicon production, with either pure DNA or disrupted cells as template, was similar for shake flask (Fig. 8a-ii) and bioreactor (Fig. 8b-ii) cultivation.

### Amplification efficiency for a mycoplasma DNA sequence largely unaffected by CHO cellular material

A common element of many approaches to absolute qPCR is the importance of amplification efficiency. To evaluate the influence of cellular material on the efficiency of amplification of a mycoplasma sequence we again prepared a pure solution of 5 ng of pPROX2 plasmid and serially diluted. To each dilution a constant volume of either water or disrupted cells from shake flask or bioreactor cultivation was added as previously. Real-time PCR was performed and Cq values plotted (Fig. 9). Between two and six tenfold dilutions (1.54 × 10^7^ copies to 1540 copies of pPROX2 plasmid) amplification efficiency is largely unaffected by the presence of disrupted CHO cells.

### LRE qPCR and SC qPCR are equivalent with respect to mycoplasma sequence quantification in the presence of disrupted CHO cells

LRE qPCR (Fig. 10a) and SC qPCR (Fig. 10b) methods of quantitation were applied to the underlying data used to generate the Cq values in Fig. 9. Both approaches resulted in reverse S-shaped curves for copy number estimation as a function of template dilution. The dotted line in both Fig. 10 graphs is an extrapolation of three spectrophotometrically measured data points and serves to aid comparison of LRE qPCR and SC qPCR data. The two methods are broadly equivalent, with disrupted CHO cells having little effect on copy number estimation over 3–6 tenfold dilutions of template material (1.54 × 10^6^ copies to 1540 copies of pPROX2 plasmid).

## Discussion

### Sample processing has a significant impact on e-pPCR of CHO cells

The purpose of PCR sample preparation is to remove inhibitors that could lead to false positives, false negatives or inaccurate quantification. Sample preparation procedures tend to significantly extend assay duration so that live or at-line data set capture is not possible. This in turn delimits application of PCR approaches in statistical process ‘quality by design’ (QbD) optimisation procedures such as design of experiments (Sadowski et al. 2015). To address these issues of standardisation and sample preparation we sought to test the following hypotheses for both e-pPCR and qPCR: (i) that the removal of cellular material may not be necessarily required for certain PCR-based assays and (ii) that the LRE qPCR method, which incorporates a putatively universal standard which is therefore potentially of use to synthetic biologists, is equivalent to conventional SC qPCR in terms of sensitivity and accuracy.

We designed MIQE-compliant primers (Bustin et al. 2009) to amplify a sequence present as a single copy within the CHO genome and a mycoplasmal sequence common to many mycoplasma species known to infect mammalian calls. We also used the primers that comprise the CAL1 calibration reaction for the LRE qPCR method (Fig. 1). We then cultivated CHO cells in shake flasks and in a rocked bag bioreactor (Fig. 2). For detection of a genomic target we serially diluted both cells and target in parallel and determined an LOD by e-pPCR of 0.2 genome copies for purified gDNA, which was increased to 2 genome copies by the presence of cell sonicates (Fig. 3). For detection of a mycoplasmal sequence by e-pPCR, we serially diluted target plasmid DNA in the presence of a constant amount of cells to mimic the early stages of a mycoplasmal contamination. An LOD by e-pPCR of 154 copies (50 pg gDNA) for purified gDNA was increased to 1540 copies (5 fg pDNA) by the presence of cell sonicates (Fig. 8). This indicates clearly that sample preparation is required for accurate and sensitive use of e-pPCR as a detection method. False positives were not observed. Cellular material might interfere with e-pPCR due to competitive binding to DNA by the many types of nucleic acid-binding proteins present in CHO cells (Baycin-Hizal et al. 2012). DNA binding by denatured proteins that persists during the conditions of PCR could also contribute to the observed inhibiton of e-pPCR by cellular material. Cellular material from shake flask or biorector cultivation had a similarly inhibitory effect on e-pPCR when used to detect a seeded plasmid encoding a mycoplasmal sequence (Fig. 8). However, when e-pPCR was used to detect a genomic sequence (Fig. 3), bioreactor-derived cellular material caused greater inhibition overall compared to shake-flask derived material. Although cell numbers were normalised for the e-pPCR experiments in Fig. 3, the bioreactor-derived cells had grown to a fourfold higher density than shake-flask-derived cells (1 × 10^7^ vs 2.5 × 10^6^ cells/mL) at the point when samples were taken. This difference in environment may have been reflected in a difference in the physiological status of the cells, with a further consequence of this being a greater DNA-binding potential of the disrupted cellular material from bioreactor cultivation.

### Sample processing has a surprisingly low order of impact on qPCR of CHO cells

Unlike e-pPCR, qPCR data collection occurs during the reaction, thus making any sample preparation time a larger fraction of total assay throughput time. We determined the extent to which the presence of disrupted CHO cells effects amplification efficiency—a key metric for multiple statistical approaches to analysis of quantitative real-time PCR data. The presence of cellular material from shake flasks had no effect on genomic target amplification efficiency profile (Fig. 4a) whereas material from bioreactors did constrict the range of reactions for which acceptable amplification efficiency was observed (Fig. 4b). This indicates that, for many commonly used qPCR methods, only minimal and rapid sample preparation is required for samples taken in the early, seed train, stages of industrial CHO cell cultivation. For amplification of a mycoplasmal sequence (Fig. 9), the presence of CHO material originating from shake flask or bioreactor cultivation influenced amplification efficiency only when greater than 1.54 × 10^7^ or less than 1500 copies of the plasmid were present in the reaction.

### The CAL1 reaction shows promise as a synthetic biology standard

As the number of innovative approaches to mammalian cell genome and gene network implementation expands (Kononenko et al. 2015) so the need for standards in synthetic biology also becomes more acute in order that variations between laboratories are reduced (Kelly et al. 2009; Beal et al. 2016). Industrial application of synthetic biology also requires standards that enable regulatory compliance and accurate analysis of chassis and bioprocess performance (Clementschitsch and Bayer 2006).

LRE qPCR, as reported by Rutledge and Stewart (2010), features the CAL1 reaction for calibration, which consists of lambda bacteriophage genome as target and a high-performance primer pair (Tables 1, 2; Fig. 1). By contrast for SC qPCR the primer pair and target sequence under investigation are normally used as the standard curve (Pabinger et al. 2014). Quantification from a universal reference standard such as CAL1 could introduce far greater reproducibility of absolute qPCR data across facilities and could in future be coupled to absolute of fluorescence meaurement standards (Würth et al. 2013) for even greater accuracy and reproducibility in sequence-specific DNA quantitation. As such we the suggest adoption of CAL1 reaction as a qPCR standard represents an excellent opportunity to improve standardisation within the synthetic biology and biotechnology communities. Standardisation between qPCR assays currently extends only to experimental setups, information reporting, such as the MIQE guidelines, and testing of food and water sources for contaminants.

We compared the accuracy of LRE qPCR and conventional SC qPCR for quantification of a genomic target sequence by juxtaposing both methods with spectrophotometric data (Fig. 5) and by statistical head-to-head analysis (Fig. 6). LRE qPCR matched the performance of conventional SC qPCR for this target. The equivalence of LRE qPCR and SC qPCR could also be seen for quantification of a mycoplasmal sequence, comparing each method to spectrophotometric data (Fig. 10). This was the case both in the presence and absence of disrupted CHO cells derived from cultivation up to 1 × 10^7^ cells/mL. When used to measure the amount of a genomic target (Fig. 7), LRE qPCR data matched spectrophotometric measurements in the presence of up to 5.5 × 10^4^ cells from shake flask cultivation and 4 × 10^3^ cells from bioreactor cultivation.

## Conclusions

We suggest that sample preparation is necessary for e-pPCR as a detection tool for CHO cell and mycoplasmal sequences. For qPCR analysis, a simple and rapid processing step, with no DNA purification, followed by 1–2 dilutions, can be sufficient to gain accurate target quantification. Finally, we suggest the LRE qPCR and the putatively universal CAL1 reaction should be tested in further contexts by the synthetic biology community with a view to possible adoption as a standard.

## Abbreviations

CHO: Chinese Hamster Ovary
PCR: polymerase chain reaction
LRE: linear regression of efficiency
qPCR: quantitative PCR
e-pPCR: end-point PCR
RT: reverse transcriptase
Cq: quantification cycle
SC: standard curve
PAT: process analytical technology
DO: dissolved oxygen
LOD: limit of detection
GAPDH: glyceraldehyde 3-phosphate dehydrogenase
